# Systems modeling of a phenazine-producing *Escherichia coli* bio-battery anolyte

**DOI:** 10.1371/journal.pone.0347163

**Published:** 2026-04-27

**Authors:** Simon M. King, Michael R. King

**Affiliations:** 1 Department of Chemical and Biomolecular Engineering, Rice University, Houston, Texas, United States of America; 2 Department of Bioengineering, Rice University, Houston, Texas, United States of America; Indiana University Purdue University Indianapolis (IUPUI), UNITED STATES OF AMERICA

## Abstract

Bioelectrochemical systems that combine living microbes with electrochemical devices are emerging as platforms for sustainable energy and chemical production. Phenazine-based organic redox flow batteries (RFBs) already achieve high-capacity, long-lived negolytes, but their performance is limited by electrolyte degradation. Recent work has demonstrated that genetically engineered, phenazine-producing *Escherichia coli* can regenerate degraded anolyte species in an operating RFB, suggesting a new class of “bio-batteries.” However, there is little quantitative understanding of how microbial physiology, phenazine toxicity, and electrochemical operating conditions jointly constrain performance. Here we develop a zero-dimensional mechanistic model that couples an engineered *E. coli* chassis to a phenazine-based flow battery half-cell. The microbial module includes Monod-type substrate uptake, growth-associated phenazine biosynthesis, first-order phenazine degradation, and a Hill-type toxicity term informed by pyocyanin’s micromolar-scale inhibitory effects. The electrochemical module computes Nernstian anode potentials from reduced/oxidized phenazine, adds a lumped area-specific resistance, and explicitly simulates charge discharge cycles with voltage cut-off criteria. We then sweep phenazine production strength, initial biomass loading, toxicity, and mediator stability to map the design space. The model predicts a strong trade-off between instantaneous current density and long-term capacity: high phenazine production and biomass can briefly reach 1.5 × 10^−3^ mA·cm ⁻ ² current densities but rapidly trigger toxicity-driven collapse, whereas more conservative designs deliver low current over an extended time period. When embedded in a simple RFB cycling protocol, moderate-production designs converge to a quasi-steady discharge capacity that is far below that of state-of-the-art phenazine RFBs, but exhibit partial self-regeneration of mediator capacity across cycles. Our results quantify the fundamental constraints of phenazine-based microbial anolytes and highlight engineering priorities, such as enhanced host tolerance and low-toxicity mediators, required for bio-batteries to become competitive or to occupy niche low-power, self-regenerating roles.

## 1. Introduction

Bioelectrochemical systems (BES) and microbial electrochemical technologies (MET) combine living cells with electrodes to convert chemical energy into electrical energy, drive reduction reactions, or synthesize value-added products [1–4]. Classical examples include microbial fuel cells and microbial electrolysis cells, in which electroactive biofilms catalyze anodic oxidation or cathodic reduction using organic substrates or waste streams [1,3]. Over the past decade, BES research has expanded from power generation towards microbial electrosynthesis, wastewater treatment, and integrated bioreactors, but scaling these systems to practically relevant power densities and long lifetimes remains a central challenge [2,5].

In parallel, redox flow batteries (RFBs) have emerged as promising technologies for grid-scale energy storage, offering decoupled power and energy, long cycle life, and flexible sizing [6–8]. Concerns over the sustainability, end-of-life management, costly recycling processes, and material intensity of conventional battery technologies have reinforced interest in alternative electrochemical platforms for niche applications, including bioelectrochemical systems and biobatteries [9–11]. Aqueous organic RFBs (AORFBs) are particularly attractive because organic redox-active molecules can be synthetically tuned for potential, solubility, and stability [6,12,13]. Phenazine derivatives have gained attention as highly soluble, reversible negolytes capable of high capacities at moderate concentrations [6,8,14]. Many phenazine-based electrolytes suffer from degradation pathways such as tautomerization and side-chain cleavage, especially at low redox potential, which limit lifetime and increase maintenance costs [7,8,15].

A recently proposed strategy to mitigate electrolyte degradation is to place living, genetically engineered microbes directly in the anolyte so that they can “repair” or regenerate degraded redox-active species during operation. Simoska and co-workers reported an RFB in which a phenazine-producing *Escherichia coli* strain regenerates degraded phenazine anolyte, markedly improving cycling stability [16]. Their work established a proof of concept for biological anolyte regeneration and motivated the broader idea of bio-batteries in which microbial metabolism maintains or adjusts electrolyte composition in situ. This approach complements earlier BES concepts where electroactive biofilms produce current directly and points to a hybrid device class blending flow-battery architectures with living catalysts [3,5].

Microbial electrochemistry has shown that microbial physiology and metabolite toxicity strongly limit current production and stability in BES [1–3]. Phenazines exemplify this dual role. In *Pseudomonas aeruginosa*, the phenazine pyocyanin functions as a redox-active virulence factor that generates reactive oxygen species and damages both microbes and host tissues [17–19]. In planktonic and biofilm cultures, pyocyanin exhibits micromolar minimum inhibitory concentrations against *E. coli* and other Gram-negative bacteria, with reported Minimum Inhibitory Concentration (MIC) values in the tens of µg·mL ⁻ ¹ (tens of µM) range [20,21]. Thus, a microbial chassis that produces phenazines to sustain an RFB also faces a risk of self-poisoning, implying an intrinsic trade-off between mediator production and cell viability.

Systems biology and genome-scale modeling offer natural tools to analyze such trade-offs. The *E. coli* genome-scale metabolic model iML1515 and its successors encode thousands of reactions and metabolites and can quantitatively predict growth and flux distributions under diverse genetic and environmental conditions [22–24]. Recent work has started to integrate thermodynamic and kinetic constraints, as well as machine learning, to capture enzyme and pathway limitations more accurately [23,25]. However, these models rarely include explicit electrochemical boundary conditions or mediator speciation, and they are not typically linked to stack-level RFB operation. Conversely, many RFB modeling studies treat electrolyte composition and degradation phenomenologically, without resolving underlying microbial processes that could regenerate or reshape the anolyte [6,7].

There is therefore a conceptual gap: we lack mechanistic yet tractable models that jointly describe (i) microbial growth and mediator production, (ii) mediator toxicity and stability, and (iii) electrochemical response and cycling in a flow-battery-like configuration. Such a model would enable systematic evaluation of design variables, including mediator production rate, biomass loading, toxicity threshold, degradation kinetics, electrode area, and ohmic resistance, and provide quantitative guidance on whether bio-batteries can approach the power and energy density of conventional AORFBs or instead occupy lower-power, self-regenerating niches. A structural comparison of representative BES, systems-biology, and RFB modeling frameworks is provided in **Table 1** to clarify the niche addressed by the present formulation.

**Table 1:** Comparison of representative BES, systems-biology, and RFB modeling framework.

Model class	Typical assumptions	Representative state variables	Strengths / applicable scope	Key limitations relative to the present work
Classical BES / MFC reactor models [26]	Often zero-dimensional or compartment-based; may assume well-mixed bulk phases; current generation typically linked to substrate oxidation and electrode kinetics; frequently focused on electroactive biofilms	Biomass or biofilm density, substrate concentration, mediator concentration (if included), current, voltage	Useful for analyzing microbial current generation, substrate conversion, and reactor-level BES performance	Usually do not represent soluble phenazine regeneration in an RFB-like anolyte or explicit charge–discharge cycling
Genome-scale / systems-biology models of E. coli [27]	Large metabolic networks with steady-state flux balance or constraint-based assumptions; often omit explicit extracellular electrochemistry	Intracellular fluxes, metabolite exchange rates, biomass growth	Capture metabolic capabilities, pathway bottlenecks, and genetic interventions in detail	Typically do not include mediator redox speciation, toxicity-driven device collapse, or battery-level cycling behavior
Zero-dimensional RFB / AORFB models [38]	Well-mixed electrolyte tanks or half-cells; Nernstian potentials with lumped ohmic/kinetic losses; degradation often treated phenomenologically	Reduced/oxidized species concentrations, cell voltage, current density, capacity	Useful for predicting voltage, capacity fade, and cycling response in flow-battery systems	Do not resolve microbial growth, substrate consumption, or biological mediator regeneration
Present bio-battery model	Zero-dimensional, well-mixed anolyte; coarse-grained microbial kinetics coupled to soluble phenazine redox chemistry and simplified RFB half-cell operation	Biomass, substrate, reduced phenazine, oxidized phenazine, total phenazine, current density, voltage, discharge capacity	Designed to capture the coupled effects of microbial growth, phenazine production, toxicity, degradation, redox speciation, and cycling in a tractable hybrid BES/RFB framework	Does not resolve spatial gradients, biofilm structure, or genome-scale intracellular metabolism

We address this gap by developing a zero-dimensional mechanistic model of a phenazine-producing *E. coli* anolyte coupled to a simplified phenazine-based RFB half-cell. The microbial module consists of Monod-type substrate uptake and biomass growth, growth-associated phenazine biosynthesis with an optional non-growth term, first-order phenazine degradation, and a Hill-type toxicity function parameterized to reflect micromolar-scale inhibition by pyocyanin and related phenazines in non-native hosts [17,18,20]. The electrochemical module uses a Nernst equation to relate reduced and oxidized phenazine concentrations to anode potential, includes a lumped area-specific resistance, and computes current density from phenazine oxidation at the electrode [6,7]. We then embed this module in a simple charge-discharge protocol with a voltage cut-off, allowing direct simulation of capacity vs. cycle number for different microbial designs.

Using this framework, we systematically explored how phenazine production strength, initial biomass, toxicity threshold, and mediator stability affect: (i) instantaneous current density, (ii) cumulative charge passed before toxicity or depletion, and (iii) long-term cycling performance. We compare the resulting current densities and capacities to reported values for phenazine-based AORFBs and discuss the extent to which engineered bio-batteries can realistically compete with or complement purely synthetic systems [6,8,15,28]. Finally, we use the model to identify specific biological and electrochemical targets such as increased phenazine tolerance, lower-toxicity mediators, and optimized electrode areas, which would be needed to close the performance gap. The accompanying SBML representation of the microbial phenazine subsystem is intended as a reusable component for future multiscale models integrating genome-scale metabolism, mediator chemistry, and device-level operation.

## 2. Materials and methods

### 2.1. Model overview

We developed a zero-dimensional, mechanistic model of an engineered *Escherichia coli* strain producing a soluble phenazine redox mediator that serves as the negolyte in a redox-flow-type bio-battery. The model couples microbial growth, substrate uptake, phenazine biosynthesis and toxicity, and first-order phenazine degradation to a simple electrochemical description of the anolyte, including Nernstian potential, ohmic losses, and explicit charge-discharge cycling.

The microbial module is intentionally coarse-grained from genome-scale reconstructions of *E. coli* metabolism such as iML1515 by aggregating central carbon metabolism into effective kinetic rates. Phenazine speciation and anode behavior are informed by recent phenazine-based negolytes and anolytes designed for aqueous and non-aqueous redox flow batteries. The overall bioelectrochemical configuration is consistent with the broader literature on bioelectrochemical reactors and zero-dimensional RFB cells and stack models.

All state variables are defined per unit anolyte volume: biomass concentration X [gDW·L ⁻ ¹], extracellular substrate S [mmol·L ⁻ ¹], reduced phenazine $\left[{\text{P}}_{\text{red}}\right]$ [mmol·L ⁻ ¹], oxidized phenazine $\left[{\text{P}}_{\text{ox}}\right]$ [mmol·L ⁻ ¹], and total phenazine $P_{\text{tot}}=[{\text{P}}_{\text{red}}]+[{\text{P}}_{\text{ox}}]$. Time is measured in hours.

### 2.2. Microbial growth and substrate uptake

Substrate uptake is modeled with a Monod-type expression for the specific uptake rate $q_{S}$ [mmol·gDW ⁻ ¹·h ⁻ ¹]:


$$q_{S}=q_{S,max}\frac{S}{K_{S}+S}$$

where $q_{S,\text{max }}$ is the maximum specific uptake rate and $K_{S}\;$ is the half-saturation constant. Reported aerobic glucose uptake rates and yields for *E. coli* were used to select $q_{S,max}$ = 10 mmol gDW ⁻ ¹ h ⁻ ¹ and a biomass yield $Y_{X/S}$ = 0.09 gDW mmol ⁻ ¹, corresponding to ~0.5 gDW per g glucose under aerobic conditions [29].

The base specific growth rate $\mu_{\text{base}}$[h ⁻ ¹] is given by:


$$\mu_{base}=Y_{X/S}q_{S}$$

Biomass and substrate balances are:


$$\frac{dX}{dt}=\mu X,\;\frac{dS}{dT}=-q_{S}X$$

where μ  is an effective growth rate that includes phenazine toxicity (Section 2.3).

### 2.3. Phenazine production, degradation, and toxicity

Phenazine production is described by a Luedeking-Piret-type rate that is primarily growth-associated:


$$r_{P,prod}=(\alpha P\mu +\beta P)X$$

where $\alpha_{P}$ [mmol·gDW ⁻ ¹] is the growth-associated coefficient and $\beta_{P}$ [mmol·gDW ⁻ ¹·h ⁻ ¹] is a non-growth-associated term. This reflects that phenazine biosynthesis in native producers is tightly coupled to central metabolism and growth, while allowing for constitutive production if desired [29,30].

Reduced and oxidized phenazine dynamics are:


$$\frac{d[P_{red}]}{dt}=r_{P,prod}-v_{elec}-k_{deg}[P_{red}]$$

$$\frac{d[P_{ox}]}{dt}=v_{elec}-k_{deg}[P_{ox}]$$

where $k_{\text{deg}}$ [h ⁻ ¹] is a first-order degradation rate capturing spontaneous phenazine degradation, side reactions, or irreversible adsorption, and $v_{\text{elec}}$ [mmol·L ⁻ ¹·h ⁻ ¹] is the electrode-mediated oxidation/reduction flux defined in Section 2.4.

Phenazines such as pyocyanin are known to be redox-active toxins that inhibit both producer and non-producer strains in the low-to-mid micromolar range [28,31,32]. To capture this, we scale the base growth rate by a Hill-type toxicity function of total phenazine:


$$\mu =\mu_{base}\;\frac{\text{1}}{\text{1}+{\left(\frac{P_{tot}}{K_{inh}}\right)}^{n_{inh}}}$$

With $K_{inh}$ = 0.01 mmol L ⁻ ¹ (10 µM) and Hill coefficient $n_{inh}$ = 2. These values were selected to produce growth inhibition of the same order of magnitude as reported pyocyanin sensitivity in non-native hosts, while allowing exploration of more tolerant designs by varying $K_{inh}$. Essentially, $K_{inh}\;$ is interpreted as an effective inhibition scale in the phenomenological toxicity term rather than as a direct one-to-one representation of a single experimentally measured MIC. Reported toxicity values for pyocyanin and related phenazines span a relatively broad range depending on the specific phenazine derivative, organism, and assay conditions. As a result, the choice of $K_{\text{inh}}=\text{1}\text{0}\;\mu \text{M}\;$ was intended as a conservative order-of-magnitude parameter representing the onset of growth suppression in a simplified model of a non-native phenazine-producing *E. coli* host, rather than as a strain-specific fitted threshold. More detailed calibration would require direct toxicity measurements for the relevant engineered strain and phenazine mixture under the intended operating conditions.

### 2.4. Electrochemical half-cell, Nernst potential and ohmic loss

We treat the anolyte as a well-mixed compartment in which the reduced phenazine is oxidized at the anode with a first-order rate constant:


$$v_{elec}=k_{elec}[P_{red}]\;$$

Where the $k_{elec}\;[h^{-\;\text{1}}]$ lumped parameter summarizes mass-transfer and heterogeneous electron-transfer limitations at the electrode. The resulting current density *j* [A·m ⁻ ²] is:


$$j=\frac{I}{A}=\frac{nF}{A}\frac{v_{elec}}{\text{1}\text{0}\text{0}\text{0}}\frac{\text{1}}{\text{3}\text{6}\text{0}\text{0}}$$

where n=2 is the number of electrons per phenazine redox event, F is Faraday’s constant (96485 C·mol ⁻ ¹), and A [m²·L ⁻ ¹] is electrode area per unit anolyte volume. The anode potential $E_{\text{an}}$ [V vs SHE] is given by the Nernst equation for a simple one-step redox couple:


$$E_{an}=\overset{\circ }{\underset{an}{E}}+\frac{RT}{nF}\text{ln}(\frac{\left[P_{ox}\right]}{\left[P_{red}\right]})$$

with standard potential $\overset{\circ }{\underset{\text{an}}{E}}=-\text{0}.\text{1}\text{0}\;$ chosen to lie within the range of reported aqueous phenazine negolytes. The cathode is represented as an ideal counter-electrode at fixed potential $E_{\text{cath}}=\text{0}.\text{9}\text{0}\;\text{V}.$ The open-circuit cell voltage is:


$$E_{oc}=E_{cath}-E_{an}$$

To account for ohmic losses, we introduce a single area-specific resistance $R_{\text{ohm}}$ [Ω·m²], representing ionic resistance in the membrane and electrolytes. The terminal cell voltage is then:


$$V_{cell}=E_{oc}-jR_{ohm}$$

This lumped description is consistent with widely used zero-dimensional RFB models and stack-level frameworks that combine Nernstian potentials with ohmic and kinetic losses. All electrochemical and cycling parameters used in the half-cell and redox-flow-battery modules are summarized in **Table 2**.

**Table 2:** Electrochemical and cycling parameters used for the bio-battery half-cell model.

Parameter	Description	Symbol	Value	Units	Notes
Electrode area per anolyte volume	Geometric electrode area normalized by anolyte volume	A	0.10	m²·L ⁻ ¹	Corresponds to ~100 m²·m ⁻ ³, representative of porous carbon felt electrodes
Electrons transferred per phenazine	Number of electrons per phenazine redox event	nₑ	2	–	Typical two-electron phenazine redox chemistry
Standard anode potential	Formal potential of phenazine couple vs SHE	E₀,_an_	−0.10	V	Within reported range for aqueous phenazine derivatives
Cathode potential	Fixed cathode formal potential vs SHE	E_cath_	0.90	V	Representative aqueous catholyte
Area-specific ohmic resistance	Lumped ionic and electronic resistance	R_ohm_	0.50	Ω·m²	Used directly in V = E − jR
Minimum discharge voltage	Voltage cut-off for terminating discharge	V_min_	0.60	V	Prevents unrealistic deep polarization
Charging current density	Constant imposed current during recharge	j_charge_	0.02	A·m ⁻ ²	Low current to avoid perturbing microbial state
Temperature	Operating temperature	T	298.15	K	Used in Nernst equation
Faraday constant	Faraday constant	F	96485	C·mol ⁻ ¹	Physical constant
Gas constant	Universal gas constant	R	8.314	J·mol ⁻ ¹·K ⁻ ¹	Used in Nernst equation

### 2.5. Charge-discharge protocol and cycle definition

To study long-term performance, we implemented an explicit charge/discharge protocol. During discharge, the current density is determined by electrochemical oxidation of the reduced mediator pool via $v_{\text{elec}}=k_{\text{elec}}[P_{\text{red}}]$, and the coupled ODE system is integrated until either a fixed maximum discharge time $t_{d}\;$ is reached or the cell voltage drops below a prescribed cut-off $V_{\text{min}}$ (typically 0.6 V), analogous to cut-off criteria in redox-flow battery cycling tests. The discharge capacity per cycle $Q_{k}$ [A·h·m ⁻ ²] is computed as:


$$Q_{k}=\int_{t_{k,start}}^{t_{k,end}}j(t)dt$$

During recharge, we impose a constant charging current density $j_{\text{ch}}$ (A·m ⁻ ²) in the opposite direction, while biomass and substrate are held fixed over the short recharge interval. The imposed current is converted to an effective phenazine flux:


$$v_{elec,ch}=-\frac{j_{ch}A\text{3}\text{6}\text{0}\text{0}}{nF}\text{1}\text{0}\text{0}\text{0}$$

with the negative sign indicating reduction of $\left[{\text{P}}_{\text{ox}}\right]$ to $\left[{\text{P}}_{\text{red}}\right]$. Phenazine balances during recharge become:


$$\frac{d[P_{red}]}{dt}=-v_{elec,ch}-k_{deg}\left[P_{red}\right],\;\frac{d[P_{ox}]}{dt}=v_{elec,ch}-k_{deg}\left[P_{ox}\right]\;$$

and the Nernst and ohmic relations are evaluated at each time point to obtain $V_{\text{cell}}(t)$. Repeated application of discharge and recharge phases from the final state of the previous cycle yields capacity vs. cycle-number profiles for each microbial design.

### 2.6. Parameterization and numerical implementation

Baseline parameters were chosen from the literature where available and otherwise selected to be physiologically and electrochemically plausible for an exploratory design study. Aerobic uptake and yield parameters for *E. coli* were guided by chemostat and constraint-based modeling studies (**Table 3**) [33]. Phenazine redox properties (standard potential, solubility, and stability) were guided by recent high capacity phenazine anolyte and negolyte reports. Zero-dimensional RFB modeling literature provided typical ranges for area-specific resistance and current densities in aqueous cells. Unless otherwise noted, simulations were performed with $q_{S,max}$ = 10 mmol gDW ⁻ ¹ h ⁻ ¹, $Y_{X/S}$ = 0.09 gDW mmol ⁻ ¹, $K_{s}=\text{0}.\text{1}\;mmol\;L^{-\text{1}}$, $k_{deg}=\text{0}.\text{0}\text{1}\;h^{-\text{1}}$, $k_{elec}=\text{2}\text{0}\;h^{-\text{1}}$, electrode area density $A=\text{0}.\text{1}\;m^{\text{2}}L^{-\text{1}}$, and ohmic resistance $R_{ohm}=\text{0}.\text{5}\;\Omega {\;m}^{\text{2}}.$ Phenazine production strength $\alpha_{P}$ and initial biomass $X_{\text{0}}$ were treated as design variables and systematically varied as described below.

**Table 3:** Aerobic uptake and yield parameters for engineered E. coli used in the base model.

Parameter	Description	Symbol	Value	Units	Notes
Maximum specific substrate uptake rate	Upper bound on aerobic glucose uptake	q_S,max_	10.0	mmol·gDW ⁻ ¹·h ⁻ ¹	Consistent with aerobic E. coli
Substrate half-saturation constant	Monod constant for substrate uptake	K_S_	0.10	mmol·L ⁻ ¹	High-affinity uptake regime
Biomass yield on substrate	Biomass formed per substrate consumed	Y_XS_	0.09	gDW·mmol ⁻ ¹	≈0.5 gDW per g glucose
Phenazine toxicity threshold	Total phenazine concentration causing 50% growth inhibition	K_inh_	0.010	mmol·L ⁻ ¹ (10 µM)	Applies to P_tot_ = P_red_ + P_ox_
Toxicity Hill coefficient	Cooperativity of phenazine inhibition	n_inh_	2.0	–	Produces sigmoidal growth suppression
Growth-associated phenazine yield	Phenazine produced per unit biomass growth	α_P_	0.03	mmol·gDW ⁻ ¹	Primary phenazine production term

All ODE systems were implemented in Python (version 3.12) and integrated using an explicit Euler scheme with time step Δt = 0.01–0.05 h. For parameter sweeps, we simulated up to 200 h of operation or until phenazine concentrations exceeded a specified toxicity threshold. The microbial phenazine subsystem, including a cumulative capacity variable, was also encoded in SBML Level 2 Version 4 to enable independent reuse and extension in standard systems biology software, and is provided as a supplemental file along with the Python implementation [34–37].

### 2.7. Use of generative AI

During the preparation of this work, the authors used ChatGPT (OpenAI, model GPT-5.1 Thinking) to assist with drafting and editing sections of the manuscript, improving code structure and documentation, and supporting implementation of the computational model. ChatGPT was also used as an interactive aid during equation development and model prototyping; however, all equations, assumptions, parameter choices, code, simulations, and scientific interpretations were reviewed, validated, and finalized by the authors. After using this tool, the authors independently checked and edited all content as needed and take full responsibility for the content of this article.

## 3. Results

### 3.1. Baseline dynamics of a phenazine-producing bio-anolyte

**Fig 1:**
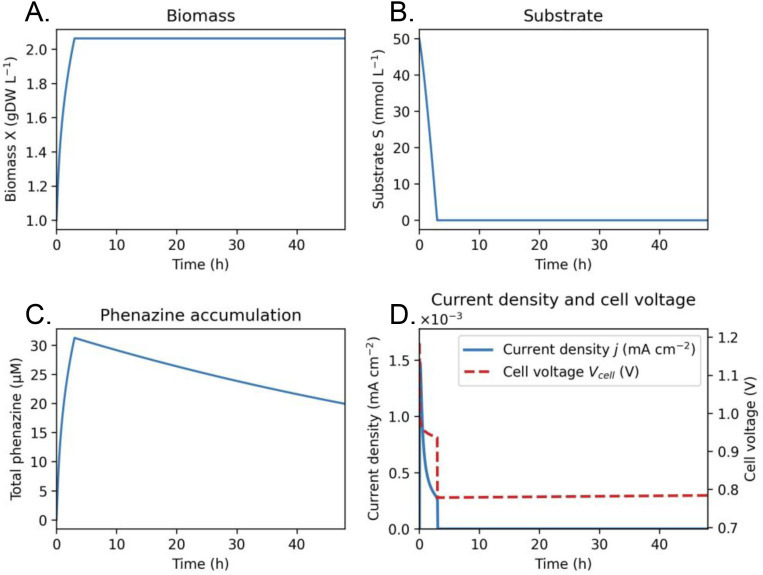
Baseline dynamics of a phenazine-producing bio-anolyte. Time evolution of (A) biomass concentration 𝐗, (B) substrate concentration 𝐒, (C) total phenazine concentration ${𝐏}_{\text{tot}}=[{\text{P}}_{\text{red}}]+[{\text{P}}_{\text{ox}}]$, and (D) current density and cell voltage for a batch culture of a phenazine-producing E. coli anolyte. Phenazine production is growth-associated and coupled to electrochemical oxidation at the anode. Current density increases with phenazine accumulation before plateauing as substrate depletion, mediator degradation, and toxicity effects become significant. Cell voltage decreases with increasing current due to ohmic losses and shifts in anode potential. Simulation parameters correspond to the baseline design described in the Methods.

We first simulated batch operation of a phenazine-producing *E. coli* anolyte to establish baseline system behavior (**Fig 1**). Biomass (E. coli) increased monotonically during the initial growth phase, accompanied by rapid substrate consumption. Phenazine is produced in the reduced form but is rapidly oxidized at the anode under baseline extraction rates, resulting in an oxidized-dominant mediator pool with only a transient reduced fraction. This regime reflects a kinetically-limited extraction process rather than accumulation of reduced mediator. As phenazine concentration increased, current density decreased to the 0.25 x 10^−3^ milliamp-per-square-centimeter range before approaching zero as substrate depletion, mediator degradation, and toxicity effects became significant. The coupled electrochemical model predicts a decline in cell voltage as ohmic losses increase with current density and the anode potential shifts positive with increasing oxidized mediator fraction.

These results establish a physically consistent baseline in which microbial growth, mediator production, and electrochemical extraction are tightly coupled, and motivate exploration of design trade-offs governing power output and operational stability.

### 3.2. Design-space exploration of phenazine production and biomass loading

**Fig 2:**
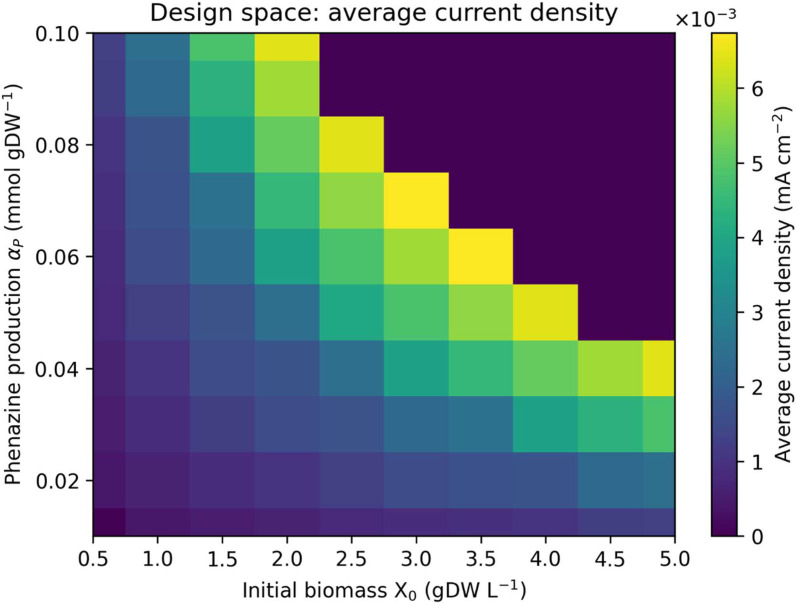
Design-space exploration of phenazine production and biomass loading. Heat map of time-averaged current density as a function of the phenazine production coefficient $\alpha_{\text{P }}$ and initial biomass concentration ${𝐗}_{\text{0}}$. Each point represents a batch simulation terminated upon reaching a phenazine toxicity threshold or the maximum simulation time. Moderate values of $\alpha_{\text{P}}\;$ and ${𝐗}_{\text{0}}\;$ maximize average current density, whereas more aggressive designs rapidly accumulate toxic phenazine concentrations and exhibit reduced effective performance. This design space highlights a fundamental trade-off between instantaneous power output and operational stability.

To identify design regimes capable of maximizing electrical output, we performed a parameter sweep over the phenazine production coefficient (αₚ) and initial biomass concentration (X₀), computing the time-averaged current density prior to toxicity-induced shutdown (**Fig 2**). Increasing either αₚ or X₀ initially enhances current density by accelerating phenazine accumulation and electrode turnover. However, beyond a moderate regime, further increases in either parameter produce diminishing returns due to rapid accumulation of toxic phenazine concentrations.

The resulting design space reveals a ridge of optimal performance at intermediate αₚ and X₀ values, highlighting a fundamental trade-off between aggressive mediator production and long-term viability. Designs optimized solely for instantaneous power density are predicted to fail rapidly, whereas more conservative designs operate for extended periods but at substantially lower current densities. In the following sections, we refer to designs with αₚ values near the center of this viable ridge as *moderate-production* regimes, and to designs near the upper bound of αₚ that maximize instantaneous current density as *high-production* regimes.

The current densities predicted here are lower than those reported for experimental *E. coli* bioelectrochemical systems. The peak modeled value in the present study is approximately 1.5 × 10 ⁻ ³ mA cm ⁻ ², whereas reported phenazine flow cells commonly operate at 20–100 mA cm ⁻ ² depending on molecular design and cycling conditions [38]. This implies that achieving comparable performance would require roughly a 4–5 order of magnitude increase in effective current-generating capacity. Within the present framework, that improvement would need to arise from combined increases in the accessible reduced phenazine inventory, the microbial rate of mediator regeneration, and the electrode-area-normalized extraction rate, together with reduced transport and ohmic limitations. The present model describes a plausible low-power microbial operating regime rather than an immediately competitive redox-flow-battery architecture. The design objective for such a system may be better understood as achieving sustained, self-regenerating electrochemical output within biologically accessible constraints, while future improvements in mediator production, microbial tolerance, and electrochemical utilization could increase performance further.

### 3.3. Coupling microbial dynamics to redox-flow battery cycling

**Fig 3:**
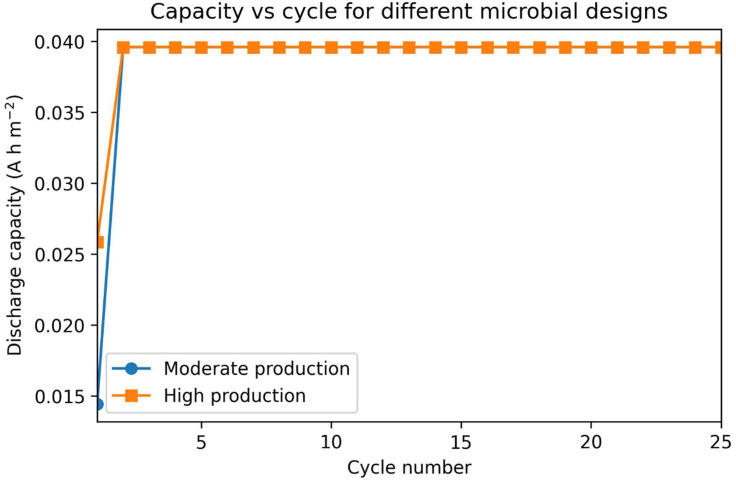
Cycling performance of microbial designs embedded in a redox flow battery model. Discharge capacity as a function of cycle number for a moderate-production design and a high-production design. Each cycle consists of a discharge step governed by Nernstian electrode potentials, ohmic losses, and a voltage cut-off, followed by a recharge step that regenerates reduced phenazine. The moderate-production design exhibits relatively stable capacity over multiple cycles, whereas the high-production design shows higher initial capacity but more pronounced capacity fade due to mediator degradation and toxicity-induced suppression of microbial activity.

**Fig 4:**
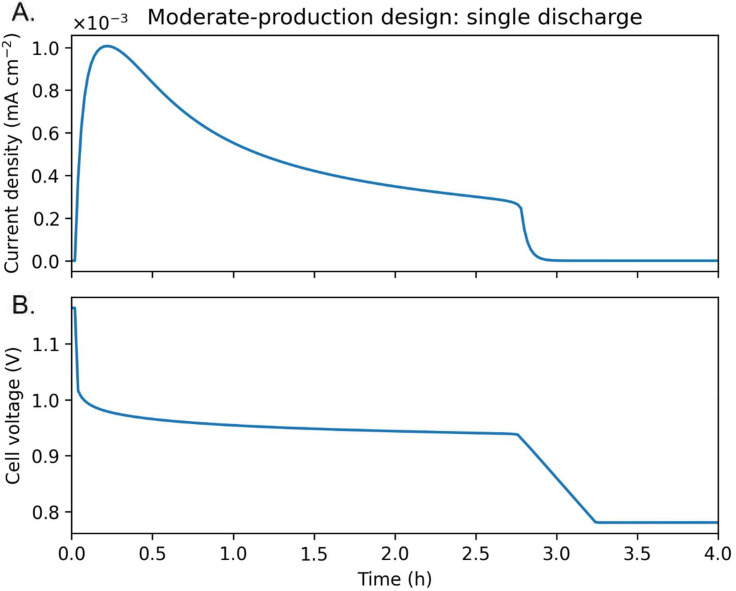
Single-cycle discharge behavior of a moderate-production bio-battery. Time evolution of (A) current density and (B) cell voltage during a single discharge cycle for the moderate-production design. Current density decreases continuously as reduced phenazine is oxidized and the anode potential shifts positive. Discharge terminates when the cell voltage reaches the prescribed cut-off, illustrating the direct coupling between microbial mediator availability and battery-level performance metrics.

We next embedded the microbial anolyte model within a simplified redox flow battery framework incorporating Nernstian electrode potentials, ohmic losses, and voltage cut-offs during discharge and recharge. The purpose of this analysis is to evaluate the internal consistency of the coupled biological and electrochemical dynamics under cycling, rather than to predict long-term durability. Following an initial transient in which mediator inventory and redox state adjust, the system converges to a steady cycling regime in which successive discharges reach the voltage cut-off at similar redox states. As a result, discharge capacity increases during early cycles and subsequently plateaus, remaining approximately constant even over extended cycling within the present model (**Figs 3**,**4**).

**Fig 3** compares cycling behavior under moderate and high mediator production rates. While higher production alters the initial transient behavior by accelerating mediator accumulation during early cycles, both designs converge to the same steady-state discharge capacity once a repeatable cycling regime is established. In both cases, capacity is not prescribed or conserved but instead emerges dynamically from the interaction between microbial mediator production, electrochemical extraction, and the imposed voltage cut-off. The convergence of capacity across cycles therefore reflects the establishment of a repeatable cycling trajectory rather than an assumption of intrinsic stability.

This steady behavior arises because the net mediator inventory per cycle stabilizes once mediator production during growth phases and electrochemical reduction during recharge collectively balance degradation losses. Capacity stability is thus an emergent outcome of the modeled dynamics and is not enforced by conservation constraints or fixed capacity assumptions. Within this mathematical framework, extending the number of simulated cycles does not induce additional capacity fade in the absence of additional time-dependent degradation mechanisms.

While first-order mediator degradation is included explicitly, the model does not capture additional time-dependent degradation mechanisms such as progressive loss of microbial activity, spatial concentration gradients, biofilm formation, or mass-transfer limitations near the electrode surface. These effects could reduce effective mediator transport, alter local toxicity exposure, and further limit electron-transfer rates, thereby changing both current output and apparent operating stability. The trends reported here should therefore be interpreted as idealized system-level behavior under well-mixed conditions, while future models should incorporate spatial transport and surface-associated microbial growth to better represent practical bioelectrochemical devices.

Single-cycle discharge profiles (**Fig 4**) demonstrate that current density decays continuously within each discharge as the reduced phenazine fraction is oxidized and cell voltage approaches the cut-off threshold. These results illustrate how microbial physiology and redox kinetics jointly determine battery-level metrics such as discharge capacity and voltage efficiency.

### 3.4. Sensitivity to phenazine toxicity and degradation

**Fig 5:**
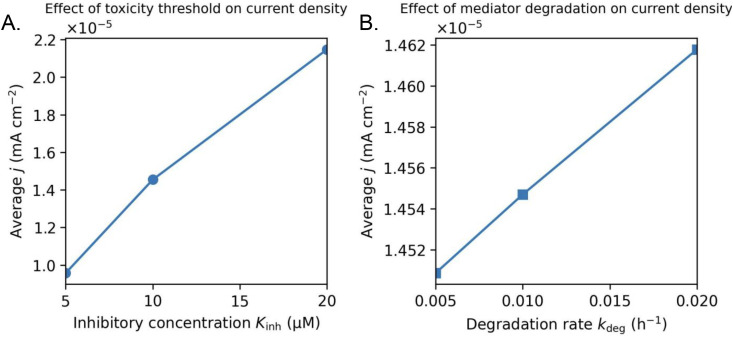
Sensitivity of average current density to phenazine toxicity and degradation. Dependence of time-averaged current density on (A) the phenazine inhibitory concentration (${𝐊}_{\text{inh}}\;$) and (B) the first-order phenazine degradation rate constant (${𝐤}_{\text{deg}}\;$) for a moderate production design. Increasing K_inh_ enhances current density by reducing toxicity-induced suppression of microbial growth and mediator production. Increasing kdeg produces a modest increase in average current density by limiting phenazine accumulation and delaying toxicity, despite faster irreversible mediator loss. These trends reflect coupled biological-chemical effects under a toxicity-limited operating protocol rather than intrinsic improvements in electrochemical efficiency.

To assess the relative influence of host tolerance and mediator stability on electrochemical performance, we performed a sensitivity analysis on the phenazine inhibitory concentration ($K_{\text{inh}}$) and the mediator degradation rate constant (${\text{k}}_{\text{deg}}$) for a moderate-production design (**Fig 5**). Two parameters in the model capture the main biological and chemical limits of the system. ${\text{K}}_{\text{inh}}\;$ represents how tolerant the bacteria are to phenazine toxicity: when phenazine concentrations approach or exceed this level, cell growth slows down and phenazine production is suppressed. In contrast, ${\text{k}}_{\text{deg}}\;$represents how quickly phenazine is irreversibly lost from the system through chemical degradation or other non-recoverable pathways, regardless of microbial activity. These parameters distinguish between limits set by microbial viability and limits set by mediator stability, allowing the model to separately assess how host tolerance and phenazine lifetime constrain bio-battery performance. Furthermore, $K_{\text{inh}}\;$ represents the phenazine concentration at which microbial metabolism becomes significantly inhibited, and $k_{\text{deg}}\;$ quantifies first-order loss of phenazine from both redox states.

Increasing $K_{\text{inh}}\;$ from 5 to 20 µM leads to a clear increase in average current density, reflecting reduced toxicity-induced suppression of microbial metabolism and more efficient electron delivery during discharge. This trend indicates that host tolerance primarily controls the achievable power output rather than the duration of operation under fixed discharge windows.

The dependence of average current density on mediator degradation rate reflects the coupled nature of mediator stability and microbial toxicity in the model. Increasing the degradation rate reduces the accumulation of total phenazine, which in turn delays toxicity-induced suppression of microbial growth and phenazine production. Microbial activity and mediator regeneration are sustained for a larger fraction of the operating window, leading to a modest increase in the time-averaged current density despite faster irreversible mediator loss. This trend arises from the interaction between degradation and toxicity rather than from improved electrochemical efficiency and should not be interpreted as an inherent benefit of mediator instability. Metrics that directly quantify stored charge or discharge capacity continue to decrease with increasing degradation, consistent with physical expectations. Because simulations are terminated when total phenazine exceeds a toxicity threshold (or when the maximum simulation time is reached), changes in $K_{\text{inh}}\;$ and $k_{\text{deg }}\;$ can affect both the time horizon over which current is produced and the resulting time-averaged current. **Fig 5** should be interpreted as a rate/viability sensitivity under a toxicity-limited operating protocol, rather than as a fixed-window comparison of power at identical discharge durations.

### 3.5. Phenazine redox speciation dynamics

**Fig 6:**
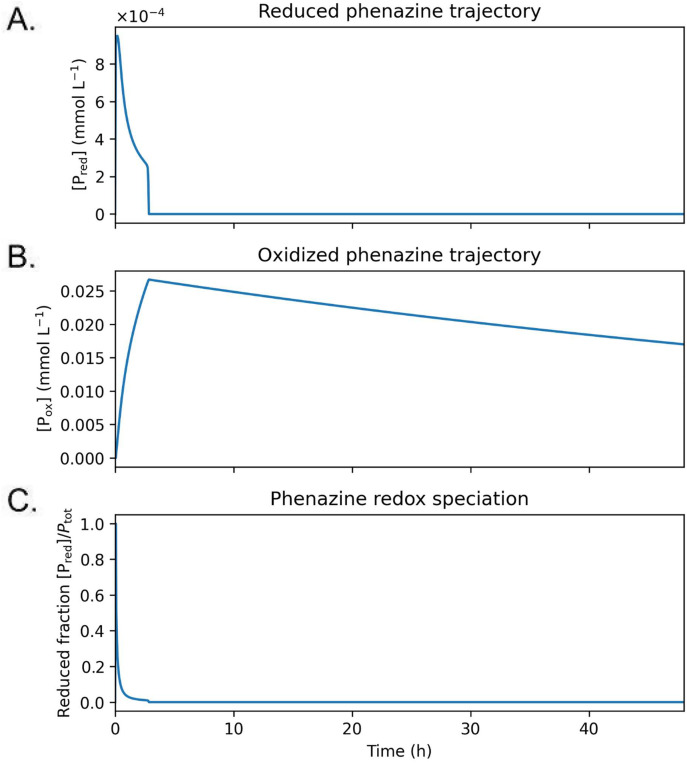
Redox speciation dynamics of phenazine during batch operation. Time courses of (A) reduced phenazine concentration $\left[{𝐏}_{red}\right]$, (B) oxidized phenazine concentration $\left[{𝐏}_{ox}\right]$, and (C) reduced fraction $[P_{red}]/P_{tot\;}$ for the moderate-production design under batch operation. Phenazine accumulates predominantly in the reduced form during early growth, with the reduced fraction remaining near unity until electrochemical oxidation and degradation become comparable to production. The evolving redox speciation directly influences anode potential, voltage utilization, and susceptibility to toxicity.

We examined the temporal evolution of reduced and oxidized phenazine pools for the moderate-production design (**Fig 6**). Following an initial transient in which reduced phenazine briefly accumulates, the mediator pool rapidly shifts toward the oxidized form: $P_{\text{red}}\;$ decreases to near zero while $P_{\text{ox}}\;$ rises quickly to a maximum and remains the dominant species for the remainder of the operating window. Consequently, the reduced fraction $P_{\text{red}}/(P_{\text{red}}+P_{\text{ox}})\;$ drops sharply within the first hours and stays close to zero thereafter, indicating that mediator oxidation at the anode outpaces net replenishment of the reduced pool under these operating conditions.

At later times, $P_{\text{ox}}\;$ gradually declines even though it is not converted back to $P_{\text{red }}$ in the model. This behavior reflects irreversible mediator loss processes represented by the degradation term (e.g., chemical decay, adsorption, or effective washout of electrochemically active phenazine), which dominate once $P_{\text{red}}$ is depleted and the oxidation flux becomes negligible. These dynamics indicate an oxidized-skewed mediator regime that limits the availability of reduced mediator for sustained discharge and constrains voltage utilization. Shifting toward a more balanced redox state would require increasing the effective reduction flux relative to oxidation (e.g., lowering current demand or current density via larger electrode area, and/or enhancing microbial electron delivery capacity), thereby maintaining a larger accessible reduced mediator fraction during operation.

## 4. Conclusions

In this study, we developed a mechanistic systems-level model that couples microbial growth and phenazine biosynthesis to electrochemical energy extraction and redox flow battery cycling. By integrating Monod-type growth kinetics, growth-associated mediator production, toxicity and degradation effects, and explicit Nernstian electrochemistry, the model provides a unified framework for evaluating the feasibility of phenazine-based bio-batteries.

Our results reveal a fundamental trade-off between power density and operational stability: aggressive phenazine production and high biomass loading can transiently achieve 1.5 x 10^−3^ milliamp-per-square-centimeter current densities but rapidly drive the system into toxicity-induced collapse, whereas more conservative designs operate for extended periods at much lower power. Sensitivity analysis identifies mediator stability and host tolerance as primary bottlenecks, with improvements in chemical robustness yielding gains in both current density and lifetime. When embedded within a simplified redox flow battery architecture, the microbial anolyte exhibits partial self-regeneration of capacity across cycles, albeit at energy and power densities below those of state-of-the-art abiotic systems.

The model is most immediately useful as a design-screening framework for identifying favorable operating regimes and prioritizing which biological and electrochemical constraints should be targeted experimentally. Future studies may validate these predictions using bench-scale bioelectrochemical systems. A phenazine-producing E. coli strain grown in an H-cell or flow-through reactor with a defined mediator concentration could be used to track current output, biomass growth, substrate consumption, and mediator stability over time under varying production strengths and substrate conditions. Such measurements would allow direct comparison with the model trajectories and help identify which parameter assumptions require refinement.

Future work with this modeling approach could include complete genome-scale metabolism managed by coupling the dynamic framework to constraint-based models like dFBA. Through this or other forms of large-scale flux analysis, this highly scalable strategy allows for the representation of intracellular metabolism using stoichiometric flux balances, bypassing the need for explicit kinetic equations for all molecular species.

The modeled bio-battery does not compete directly with conventional batteries for high-power applications, it highlights potential niches for living electrochemical systems in low-power, self-maintaining, or resource-constrained environments. The framework presented here provides quantitative guidance for engineering microbial electrochemical systems and establishes a foundation for future studies that integrate more detailed metabolic networks, spatial transport, and experimental validation.
